# Src Regulates the Activity of the ING1 Tumor Suppressor

**DOI:** 10.1371/journal.pone.0060943

**Published:** 2013-04-09

**Authors:** Lisa Yu, Satbir Thakur, Rebecca YY. Leong-Quong, Keiko Suzuki, Andy Pang, Jeffrey D. Bjorge, Karl Riabowol, Donald J. Fujita

**Affiliations:** 1 Department of Biochemistry and Molecular Biology, University of Calgary, Calgary, Alberta, Canada; 2 Department of Oncology, University of Calgary, Calgary, Alberta, Canada; 3 Southern Alberta Cancer Research Institute, University of Calgary, Calgary, Alberta, Canada; Wayne State University, United States of America

## Abstract

The INhibitor of Growth 1 (ING1) is stoichiometric member of histone deacetylase (HDAC) complexes and functions as an epigenetic regulator and a type II tumor suppressor. It impacts cell growth, aging, apoptosis, and DNA repair, by affecting chromatin conformation and gene expression. Down regulation and mislocalization of ING1 have been reported in diverse tumor types and Ser/Thr phosphorylation has been implicated in both of these processes. Here we demonstrate that both *in vitro* and *in vivo*, the tyrosine kinase Src is able to physically associate with, and phosphorylate ING1, which results in a nuclear to cytoplasmic relocalization of ING1 in cells and a decrease of ING1 stability. Functionally, Src antagonizes the ability of ING1 to induce apoptosis, most likely through relocalization of ING1 and down regulation of ING1 levels. These effects were due to both kinase-dependent and kinase-independent properties of Src, and were most apparent at elevated levels of Src expression. These findings suggest that Src may play a major role in regulating ING1 levels during tumorigenesis in those cancers in which high levels of Src expression or activity are present. These data represent the first report of tyrosine kinase-mediated regulation of ING1 levels and suggest that kinase activation can impact chromatin structure through the ING1 epigenetic regulator.

## Introduction

The INhibitor of Growth (ING) family of proteins are classified as type II tumor suppressors, and act as stoichiometric members of histone acetlytransferase (HAT) and histone deacetylase (HDAC) complexes [1]. Five ING genes, *ing1-5* are evolutionarily conserved [2] and most encode multiple splicing isoforms [3]. The first ING gene identified, human ING1, was discovered by PCR-mediated subtractive hybridization between normal mammary epithelial cells and breast cancer cells followed by a functional screen for tumorigenesis [4], [5]. The loss of ING1 caused tumor growth of pre-neoplastic mammary epithelial cells in nude mice, whereas the presence of ING1 inhibited growth and transformation [4], [5]. Human ING1 has four possible splice variants generated by alternative splicing of upstream exons of ING1 or internal initiation, and therefore each splice variant contains the conserved C-terminus and a unique N-terminus [6], [7]. Different isoforms of ING1 are involved in various chromatin modification complexes, and each has unique functions. In addition, ING1 isoforms have been suggested to play different roles in tumorigenesis. For example, inactivating one variant of ING1 in mice gave different outcomes than inactivating the whole gene [8], [9], and *in vitro* ING1b expression induces apoptosis [10] while ING1a induces senescence [11]. The ING proteins have been found to function in many biological processes and affect growth regulation, apoptosis, aging, and DNA repair, largely through their ability to regulate histone acetylation, thereby affecting gene expression [6], [7], [12], [13].

ING1b is the most highly expressed and widely studied isoform of the ING1 proteins [14], [15]. Levels of ING1b are decreased in a variety of cancers, including breast cancer [16]–[19]. Many mechanisms have been proposed for this decrease, such as downregulation of the expression of ING1 mRNA [17], [18], loss of heterozygosity (LOH) [20], [21], and hypermethylation of the ING1 promoter [22]. In addition, relocalization of ING1b from the nucleus to the cytoplasm has also been observed in various human cancers [15], [16], [23] and this relocalization has been shown to affect the functions of ING1b in cancer cell lines [24]–[26]. Clearly the expression level and the localization of ING1b protein are important for tumorigenesis; however the mechanisms involved in ING1 downregulation and relocalization or mislocalization, are still not fully understood.

The proto-oncogene, Src, is a non-receptor tyrosine kinase that plays an important role in transducing signals received through growth factor membrane receptors [27]. Increased expression and activation of Src has been observed in breast cancers [28], [29] as well as other cancers [30]–[33]. Recently, we reported that Src is able to trigger the degradation of the von Hippel-Lindau (VHL) tumor suppressor through direct phosphorylation of VHL [34]. The reduction of VHL levels resulted in increased HIF1-α levels and angiogenesis. In another study, overexpression of Src resulted in the mislocalization of RUNX3, a transcription factor that has tumor suppressor function [35]. Furthermore, in tumor cell lines where Src was activated, tyrosine phosphorylated RUNX3 was mainly detected in the cytoplasm [35].

It has long been established that Src is involved in tumor growth and metastasis by driving cell proliferation, survival, migration, and angiogenesis. These recent studies show that another mechanism whereby Src may promote cancer growth is by impeding the function of tumor suppressors, either causing the degradation or mislocalization of certain tumor suppressors. In this study, we asked whether Src could also regulate the ING1b tumor suppressor. The results demonstrate that Src is able to both physically associate with, and to phosphorylate ING1b. We found that Src decreases the stability of ING1b, and promotes ING1b relocalization from the nucleus to cytoplasm. In addition, we found that Src could functionally antagonize the ability of ING1b to induce apoptosis, suggesting that Src may promote tumour survival by reducing ING1 levels and causing ING1 relocalization.

## Results

### Src Physically Interacts with ING1

**Figure 1:**
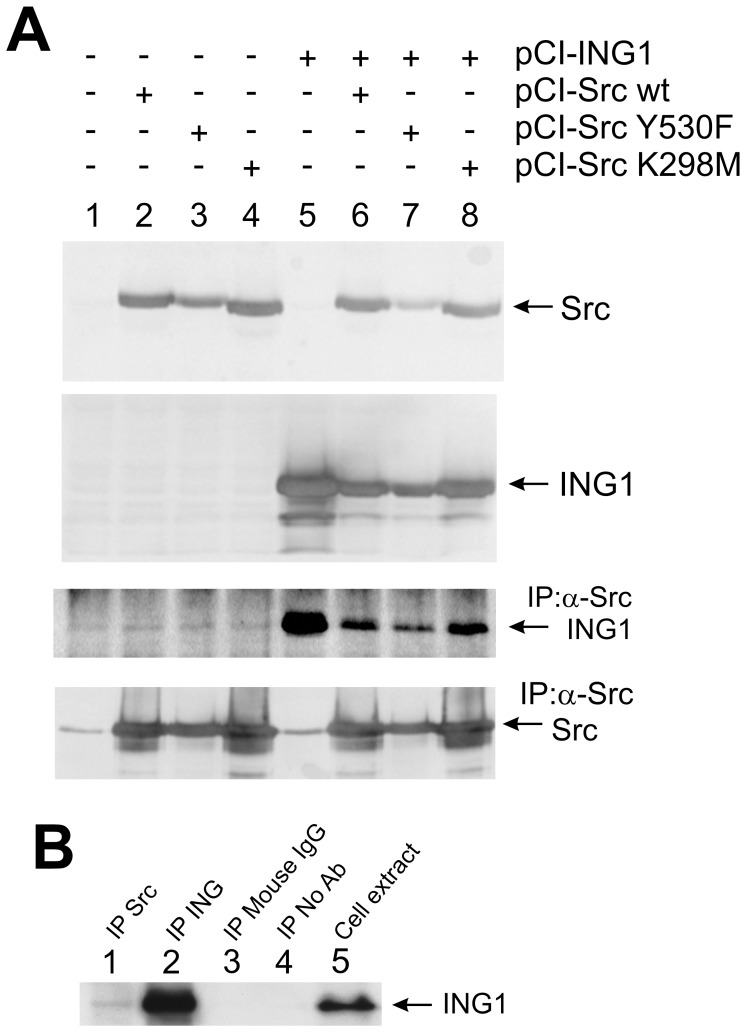
ING1 binds to Src and Src mutants. A) Wild-type Src and the mutants indicated were ectopically expressed in HEK293 cells. Expression levels of Src and ING1 are shown in immunoblots of whole cell extracts (upper two panels). Src immunoprecipitates from these cells were probed with antibodies against ING1 or Src (lower two panels). Bands at 55 and 23 kDa represent heavy and light chains of α−Src used in immunoprecipitation. The top two and bottom panels are data from the same experiment. The third panel showing ING1 co-immunoprecipitated with Src is from a separate experiment performed under the same conditions; however, a larger amount of total protein in the lysate was used for immunoprecipitation than in the bottom panel in order to aid in the visualization of endogenous ING1. Note: The level of activated Y530F Src (lanes 3 and 7, top and bottom panels) is less than that of wt or kinase-dead K298M Src because of its shorter half life. This effect is also seen in Fig. 3. B) A431 cell extracts were immunoprecipitated using either antibody against Src (lane 1) or ING1 (lane 2), using control mouse IgG antibody (lane 3), or in the absence of antibody (lane 4), followed by immunoblotting of the immunoprecipitates for ING1. Untreated whole cell extract (approx. 8% of cell extract protein used in the immunoprecipitations for lanes 1–4) (lane 5) was also blotted for ING1.

To ask if ING1 might serve as a substrate for Src, we performed immunoprecipitation-western assays to determine whether or not Src could physically interact with ING1. As shown in Figure 1A, when ING1 was expressed ectopically in HEK293 cells, ING1 was recovered in Src immunoprecipitates. Paradoxically, when ING1 was co-expressed with either WT, activated (Y530F) or kinase-dead (K295M) versions of Src, the levels of ING1 associated with Src were reduced dramatically. In addition, expression of Src also reduced the total amount of ING1 in cells. However, the reduction of ING1 that occurred with kinase-dead Src was less than the reduction observed with WT or activated forms of Src. These results suggested that increases in levels of Src could result in a decrease in total ING1 and in Src-associated ING1, in a manner that was partially, but not entirely Src kinase dependent.

Association of endogenous ING1 with endogenous Src was observed in HEK293 cells (Fig. 1A, 3^rd^ panel, lane1), and also in A431 cells (Fig. 1B, lane 1). The band intensities were lower than seen with overexpressed proteins, reflecting the relatively lower levels of these endogenous proteins.

### Src Phosphorylates ING1 *in vitro* and *in vivo*

**Figure 2:**
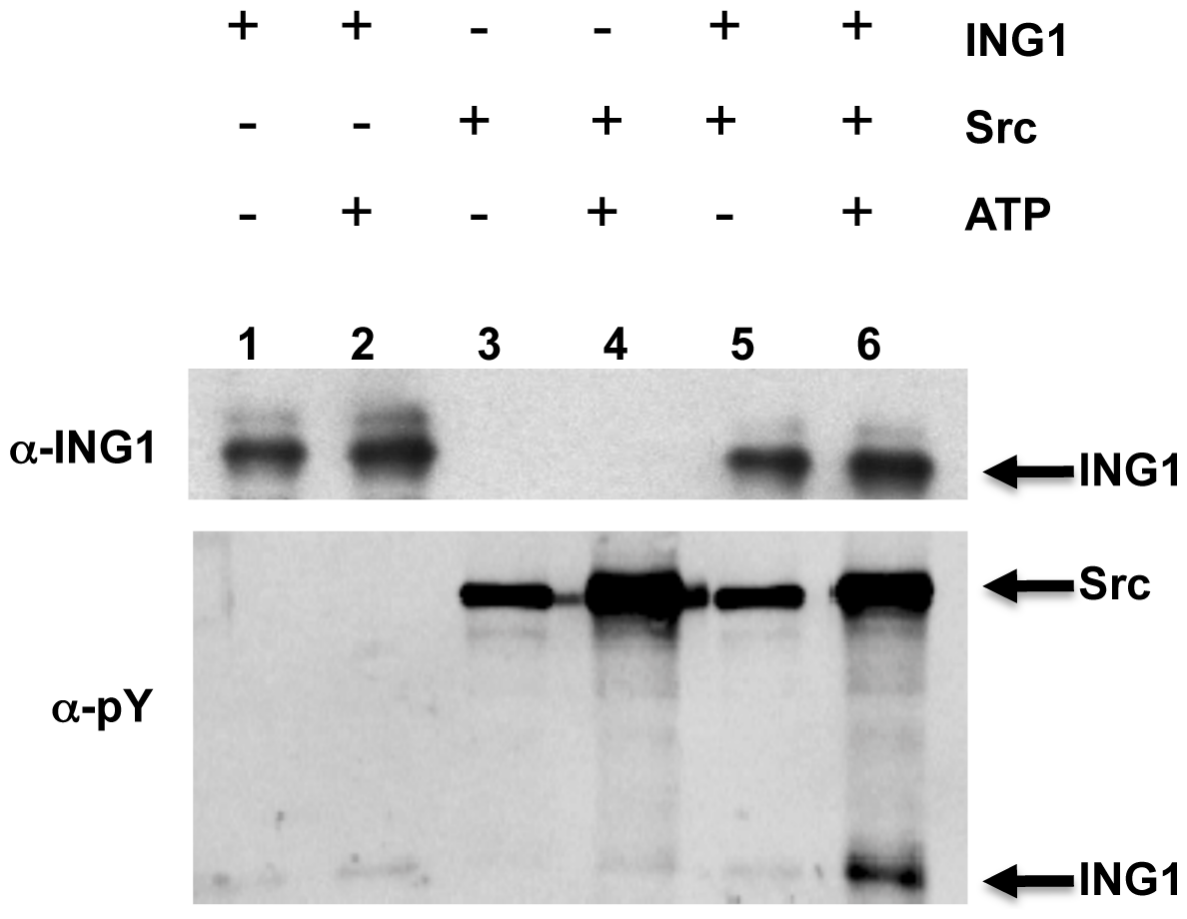
Src phosphorylates ING1 *in vitro*. ING1, Src and ATP were added to lane 6 where phosphorylation of ING1 is seen when blotting with anti-phosphotyrosine. Lanes 1–5 serve as negative controls.

**Figure 3:**
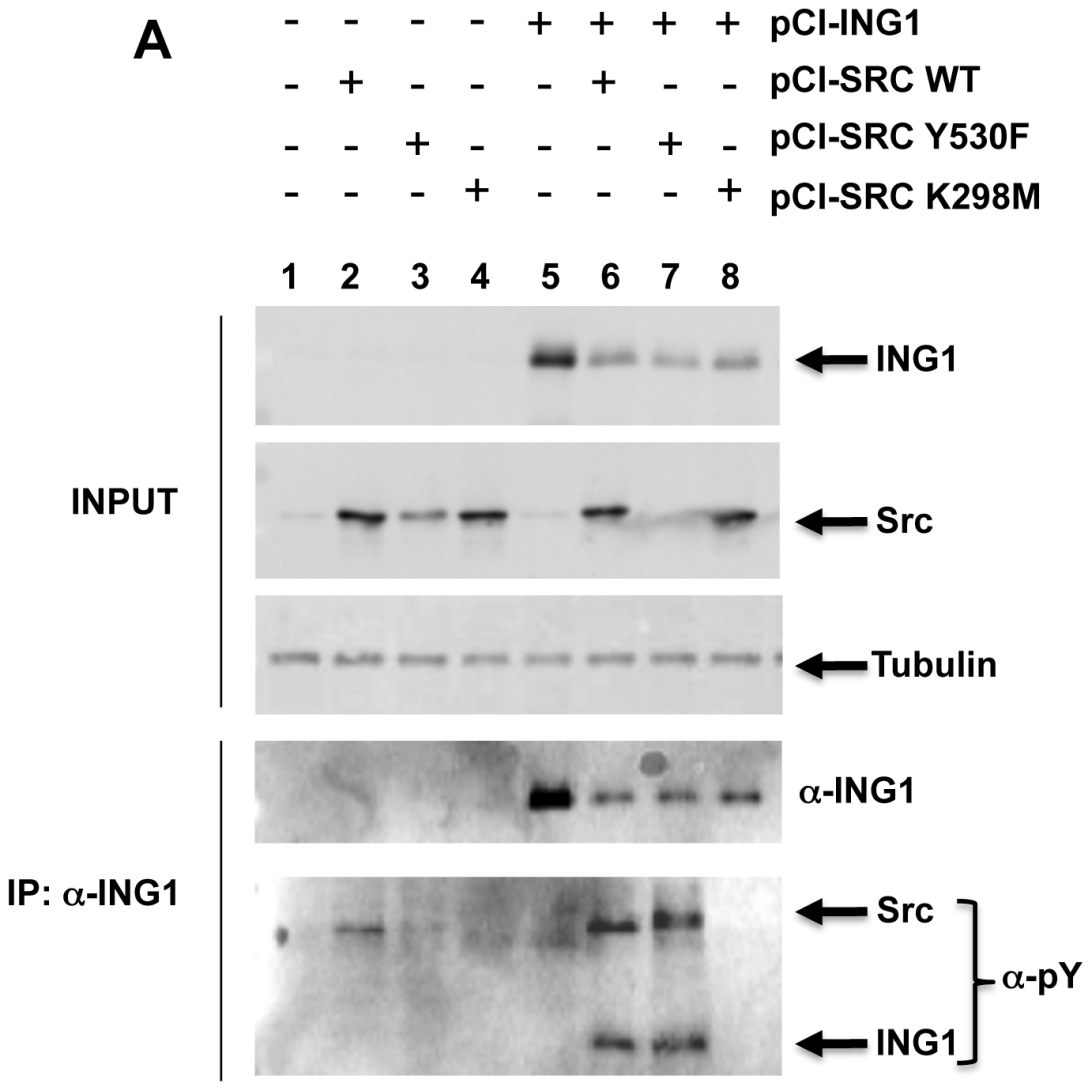
Phosphorylation of ING1 by Src *in vivo*. HEK293 cells were untransfected (lane 1), transfected with Src constructs alone (lanes 2–4) ING1 alone (lane 5) or cotransfected with ING1 plus wt Src (lane 6), activated Src (lane 7) or kinase dead Src (lane 8). Lysates (top panels) or ING1 immunoprecipitates (bottom panels) were blotted with the indicated antibodies.

To address whether the Src-ING1 interaction promoted ING1 phosphorylation by Src, an *in vitro* kinase assay was performed. Bacterial recombinant ING1 protein was resuspended in kinase buffer, and incubated in the presence of ATP, purified Src, or both ATP and Src. Only in the presence of both ATP and Src, an intense band corresponding to the size of ING1 was seen when the reaction was blotted with anti-phosphotyrosine (α-pY) antibody (Figure 2). In order to determine if ING1 was phosphorylated by Src *in vivo*, HEK 293 cells were transfected with plasmids to express ING1 alone or in the presence of ectopically-expressed WT Src, activated Src (Y530F), or kinase-dead Src (K298M). Cell extracts were immunoprecipitated with α-ING1 antibody, and blotted with α-pY antibody. As shown in Figure 3, a band corresponding to the size of phosphorylated ING1 was detected in cells expressing ING1 together with WT Src or activated Src (lanes 6 and 7, lower panel). This result confirmed that ING1b can be phosphorylated by Src *in vivo*. A decrease in total ING1 and in ING1 recovered in Src immunoprecipitates was also observed in cells expressing elevated levels of Src (Figure 3, lanes 6–8), which was consistent with results previously described in Figure 1.

**Figure 4:**
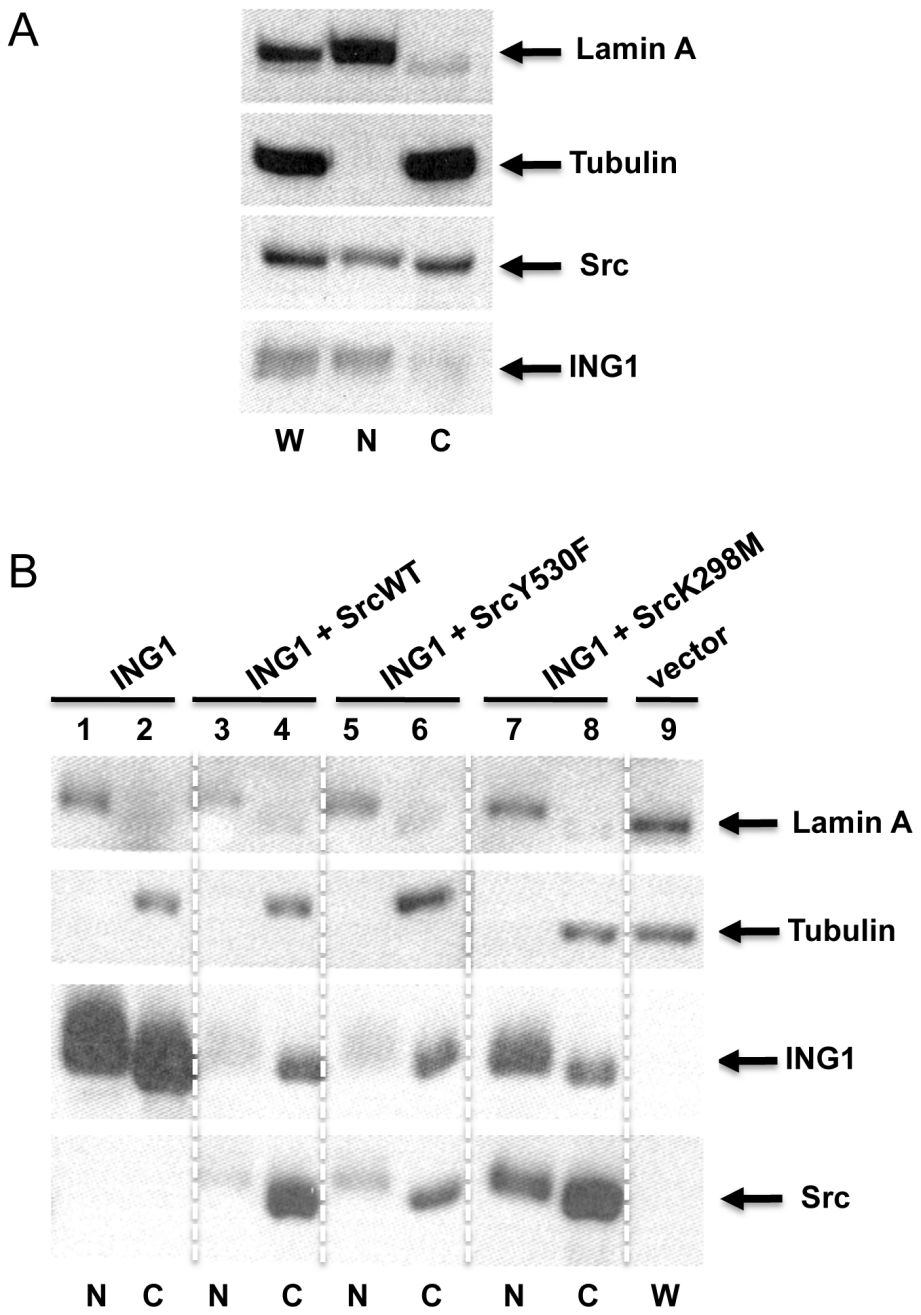
Subcellular localization of ING1 and Src. **A)** Lamin A serves as a nuclear marker while tubulin is cytoplasmic. Src is found in both the nuclear (N) and cytoplasmic (C) fractions while ING1 is primarily nuclear. W indicates whole cell lysate. **B)** ING1 localization in response to Src. ING1 was coexpressed with the Src variants noted.

**Figure 5:**
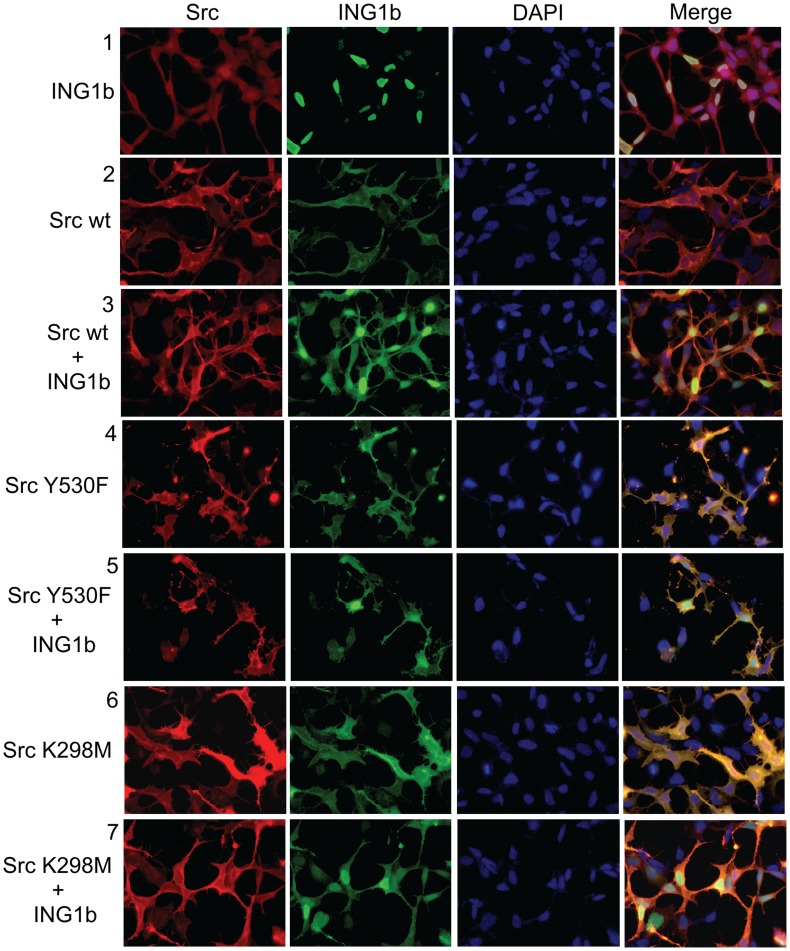
HEK293 cells were transfected with constructs expressing the indicated proteins. Row 1: ING1b; Row2: Src wild type; Row3: Src wild type+ING1b; Row4: Src activated Y530F; Row5: Src activated Y530F +ING1b; Row6: Src kinase dead K298M; Row7: Src kinase dead K298M +ING1b. Analysis by fluorescence microscopy at 24 hours post transfection utilized a primary antibody hybridoma cocktail mix of Cab 2,4,5 and 9 for ING1, and Mab327 for Src. Secondary fluorescent antibodies were Alexa Fluor 488 and Alexa Fluor 568 (Invitrogen) for ING1 and Src respectively. Hoechst 33342 was used for nuclear staining.

Since the majority of Src is located on membranes [36] but the majority of ING1 is nuclear [37], we asked if Src might have effects on subcellular localization of ING1. To examine the nuclear versus cytoplasmic localization of ING1 and Src, we prepared nuclear and cytoplasmic fractions from asynchronously growing cells using a rapid fractionation protocol [38]. As shown in Figure 4A, Src is found in both cytoplasmic and nuclear fractions with the majority in the cytoplasmic fraction, while the majority of ING1 is found in the nuclear fraction. To ask if the different forms of Src would differentially affect ING1 subcellular localization, nuclear and cytoplasmic fractions of cells cotransfected with ING1 and wild-type, activated or kinase dead Src, were examined by western blotting. As shown in Figure 4B, all forms of Src reduced ING1 levels as before. While both wild-type and activated Src resulted in elimination of ING1 from the nucleus, significant amounts of ING1 remained nuclear in cells expressing the kinase dead Src (panel 3 of Figure 4B, compare lanes 7 and 8 with lanes 3–6), suggesting that Src kinase activity was responsible for nuclear to cytoplasmic relocalization of ING1. This observation was not due to fraction cross contamination since tubulin was noted to be wholly cytoplasmic in all of the preparations. In order to further confirm these observations, we performed immunofluorescence analyses on whole cells using antibodies specific for Src and ING1B (Figure 5). Results of these experiments were consistent with those obtained through whole cell fractionation analysis (Figure 4B), as all forms of Src caused a relocalization of ING1 and kinase-dead Src did so less efficiently than wt or activated Src (Fig. 5).

### Src Decreases the Stability and Level of ING1

**Figure 6:**
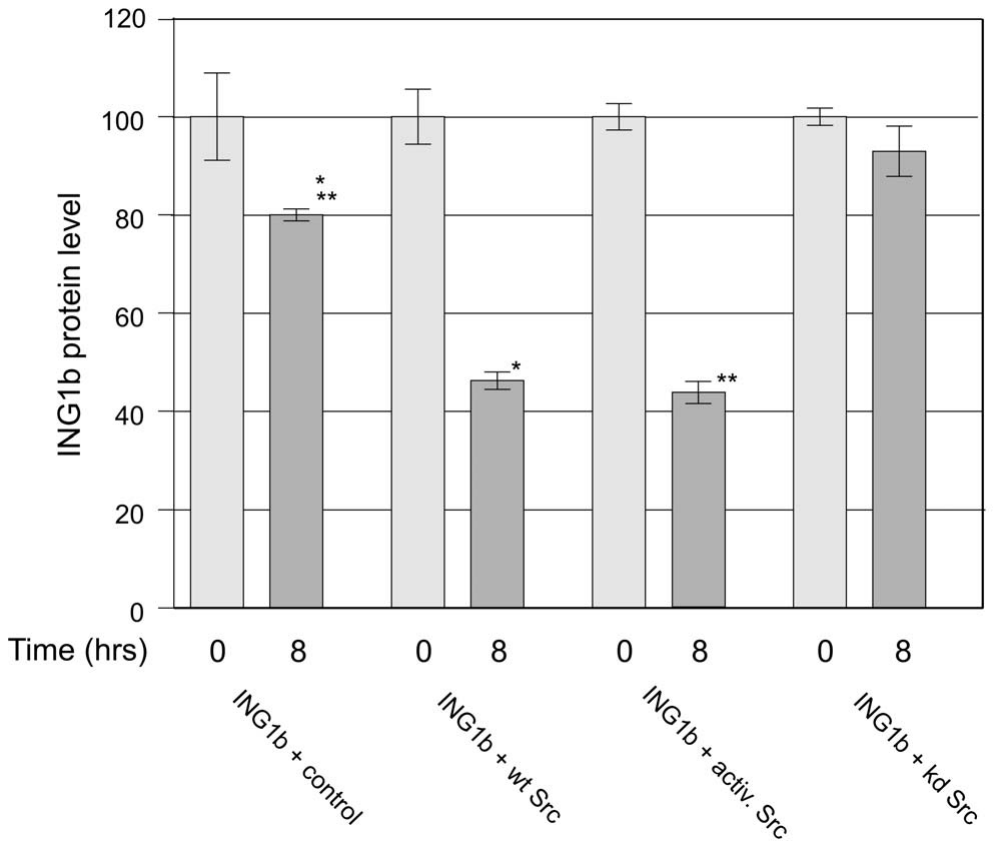
Src-dependent decrease in ING1B stability. HEK293 cells were transfected overnight with plasmid constructs expressing ING1b and either control or Src-expressing plasmids. The following morning, cycloheximide (100 ug/ml) was added to each well and the cells were harvested at the indicated times following cycloheximide addition. Cell extracts were analyzed by western blotting with anti-ING1b antibody. The ING1b bands were quantitated by scanning and the data normalized to 100% at time 0 for each condition. The results are from triplicate wells of cells +/−1 S.E. (*p<0.01, **p<0.001).

Since it was observed in the *in vivo* experiments that ING1 levels were decreased in the presence of WT, activated, or kinase-dead Src, we next asked if the various forms of Src were involved in regulating the stability of ING1. Cycloheximide (CHX) was used to block protein synthesis, and ING1 levels were analyzed at 0 time and after 8 hrs of CHX treatment by western blotting. In the absence of elevated Src 80% of ING1 protein remained at 8hrs (Figure 6) which agrees well with a previous study [39]. In the presence of wt or activated Src, the level of ING1 decreased to 45% or 42%, respectively, after 8 hours, whereas very little decrease was observed in the presence of kinase-dead Src. This indicated that Src reduced the stability of ING1 significantly and suggested that Src-induced ING1 destabilization was largely kinase-dependent. This was further supported by the results of cell fractionation experiments in which the activated and wt forms of Src eliminated ING1 from the nucleus to a greater degree than kinase-dead Src (Figures 4B and 5).

### Src Inhibits ING1-induced Apoptosis

ING1b is the predominant ING1 isoform in most cells examined and it has been shown to effectively induce apoptosis when overexpressed [10], [11]. To ask if the different forms of Src reduced ING1 levels and/or activity sufficiently to interfere with ING1-induced apoptosis, ING1 was expressed in the absence and presence of Src, in the.

**Figure 7:**
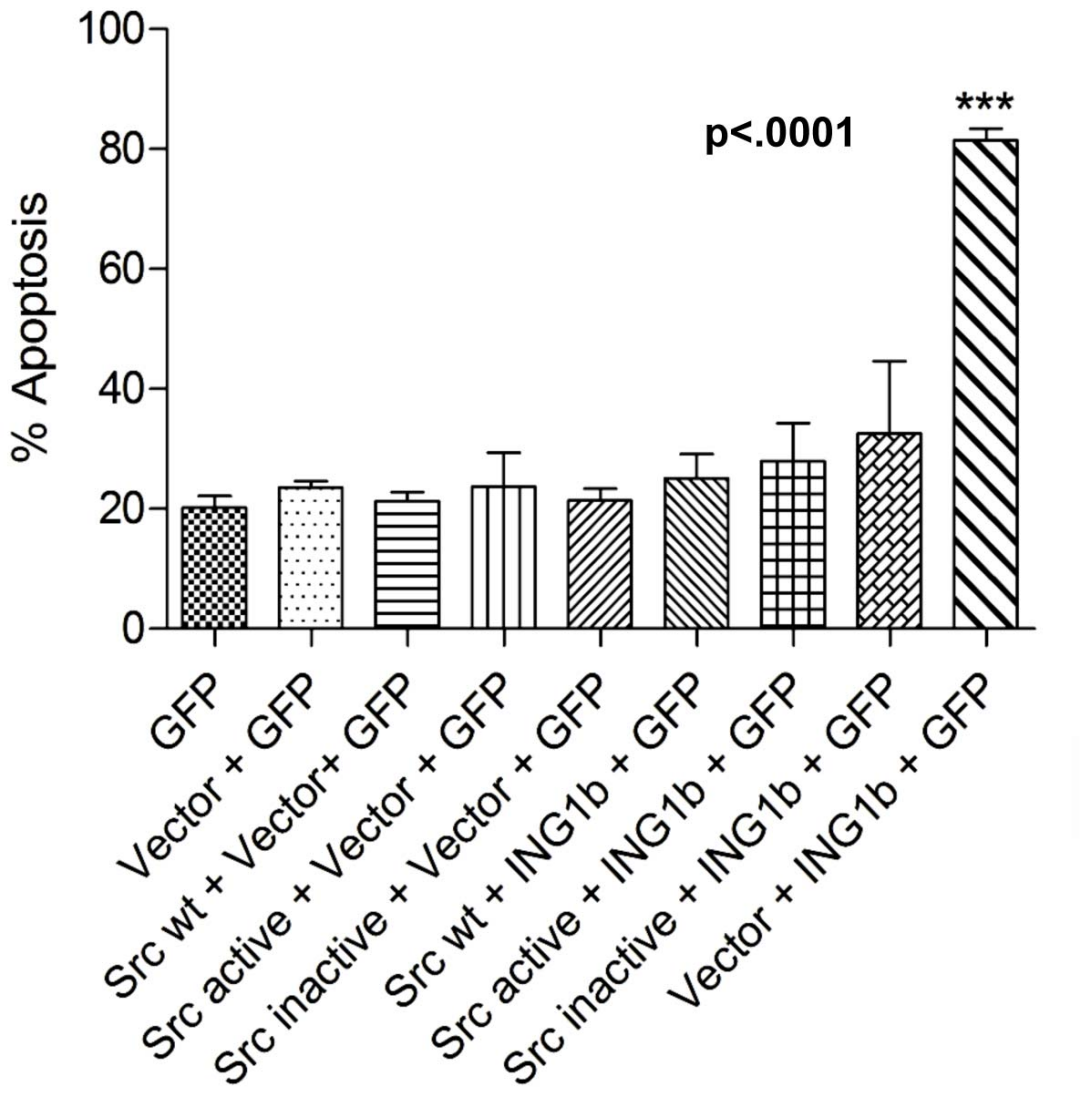
Kinase active and kinase inactive forms of Src block the ability of ING1 to induce apoptosis in MDA-MB-468 cells. Exponentially growing cells were transfected with GFP expression construct in the absence or presence of the additional constructs indicated. ING1 expression increased the level of apoptotic cells from 20% to 80% within 24 hours as estimated by Annexin V staining in flow cytometry. Active forms of Src completely blocked ING1-induced apoptosis, and kinase-inactive Src was nearly as effective in preventing ING1-induced apoptois.

MDA-MB-468 breast cancer cell line that we have shown is sensitive to ING1-induced apoptosis [40]. As shown in Figure 7, transfection with a GFP expression construct resulted in 20% of transfected cells undergoing apoptosis while GFP plus ING1 expression induced apoptosis in 80% of the cell population. Coexpression of wild-type Src with ING1 blocked the ability of ING1 to induce apoptosis. Coexpression of activated Src with ING1 also blocked ING1-induced apoptosis, even though the levels of Y530F protein expressed are considerably lower as noted in Figures 1 and 3. Although it was less effective than kinase active Src, even the kinase dead mutant of Src was able to block the majority of ING-induced apoptosis, consistent with it also reducing levels of ING1.

## Discussion

ING1 is a type II tumor suppressor whose activity affects many different *pathwa*ys, including growth regulation, apoptosis, DNA repair, chromatin remodeling, and gene expression [6], [7], [12], [13]. In this study we have shown that ING1 physically associates with, and is a target substrate of the Src tyrosine kinase *in vitro* and *in vivo*, that Src contributes to reducing levels of ING1 by phosphorylation-dependent and phosphorylation-independent mechanisms, and that such reduction blocks the ability of ING1 to induce apoptosis. This suggests that Src may contribute to regulating ING1 levels and thus act to alter cell susceptibility to undergoing apoptosis since ING1 has been reported by many groups to enhance apoptosis [9]–[11], [41]–[47].

At least two previous studies have identified sites of ING1 phosphorylation that affect ING1 function. In one study, ING1 in MMRU cells was noted to be phosphorylated at Ser-126 in response to UV, and this increased ING1 protein stability. The half-life of a FLAG-tagged ING1 protein was estimated to be ∼17 hours while mutation of the Ser-126 residue to alanine resulted in a decrease in half-life to ∼6 hours [39], [48]. A second study based upon bioinformatic identification of a consensus 14-3-3 binding site in ING1 showed that phosphorylation of Ser-199 promoted binding of 14-3-3 proteins, leading to accumulation of ING1 in the cytoplasm and the loss of ING1-induced expression of the CDK inhibitor p21. In contrast, an S199A mutant was found to be constitutive in inducing apoptosis, perhaps differentiating nuclear and cytoplasmic roles of ING1. The potential sites of ING1 phosphorylation as estimated by three independent programs (NetPhos2, KinasePhos and Motif Scan, [49]–[51]) are shown in Figure S1. Two of the three programs predict that S12*6* would be phosphorylated while all three predict that S199 would be phosphorylated. Regarding potential Src sites, two of the programs predicted that Y212 could be phosphorylated while one program predicted that Y55 was a potential site. The Y55 residue is located in a region that might interact with SAP30 of the Sin3A HDAC complex [52] while Y212 is located within the plant homeodomain (PHD) o*f ING1,* the domain responsible for specific interaction with the amino tail of histone H3 when lysine 4 is modified to H3K4Me3 [45]. Y212, which occupies a hydrophobic groove in the PHD that interacts with the trimethylated N residue of histone H3K4 has also been shown to be required for ING1 to affect apoptosis and DNA repair [45], and for induction of senescence in response to ras overexpression [53]. Since Y55 is located in the domain of ING1 that interacts with HAT and HDAC complexes and Y212 is needed for targeting of the complexes to H3K4Me3, phosphorylation of either site would be expected to affect the ability of ING1 to contribute to reading or writing of the histone code.

Numerous studies have indicated that phosphorylation can affect the stability of target proteins. This study, as well as a previous report identifying Ser-126 of ING1 as a kinase target, confirm that ING1 stability is also regulated by phosphorylation. However, the mechanism may be complex since phosphorylation of Ser-126 stabilizes the protein while phosphorylation by Src reduces ING1 stability and causes a relocalization of ING1 from the nucleus to the cytoplasm. In addition, our results indicate that increases in Src levels can also decrease levels of ING1 through both Src kinase-dependent and Src kinase-independent mechanisms. Although the exact mechanism(s) by which ING1 stabililty is regulated appear to be complex, the level of this protein in cells is likely to have significant impact since it acts as a stoichiometric member of major histone deacetylase (HDAC) complexes [1]. Consistent with this, many studies have reported that levels of the ING1 tumor suppressor decrease in breast cancers [16]–[19]. Our lab and others have found that Src levels generally increase in breast cancers [28], consistent with our current study in which Src reduces ING1 levels. One unexpected result of our study was that although Src and particularly activated Src very efficiently reduced ING1 levels and blocked ING-induced apoptosis, even the kinase-dead form of Src was able to interact with ING1 and could cause a partial reduction of ING1 levels in cells, and of ING1 stability. These kinase-independent effects of Src suggest that besides phosphorylation, physical interaction with Src may trigger degradation of ING1, or that kinase-dead Src may recruit and/or activate other tyrosine kinases to target ING1 through acting as an adaptor molecule. Kinase-independent functions of Src have been reported previously in several studies including effects on VHL [34], cell adhesion and osteoclast function [54], [55]. The major phenotype noted in Src −/− mice was a defect in osteoclast function resulting in osteopetrosis. When knockout animals had a kinase-dead version of Src added back, osteoclast function was rescued, leading the authors to speculate that this may be due to an ability of the kinase-dead Src to recruit other tyrosine kinases since tyrosine phosphorylation was restored by the kinase-dead Src [54], consistent with a requirement for intact SH2 and SH3 domains for recovery of function [55]. Thus, in the case of ING1 degradation, the presence of Src, with or without kinase activity may be enough to induce complex formation and subsequent tyrosine phosphorylation through other kinases such as the insulin receptor, the EGF receptor, Jak kinases or c-Abl. This may be likely since some of these kinases show high scores as potential kinases able to phosphorylate ING1 as noted in Figure S1.

Together with our previous report regarding Src-regulated degradation of the von Hippel-Lindau (VHL) protein that functions to inhibit vascular endothelial growth factor expression [34], this study supports a model in which Src contributes to the regulation of a subset of tumor suppressor proteins by altering their stability and half-life. This could occur through phosphorylation-induced ubiquitination as was noted in the case of the VHL protein or by physical binding of the Src protein promoting degradation via ubiquitin-linked degradation or through other mechanisms such as autophagy.

## Materials and Methods

### Cell Lines, Media, Plasmids, and Transfection

HEK293, MDA-MB-468, and A431 cell lines were purchased from American Type Culture Collection (ATCC, Manassas, VA). Cells were cultured in Dulbecco’s modified Eagle’s medium (DMEM) supplemented with 10% fetal bovine serum (FBS) and grown at 37°C with 5% CO_2_. Src plasmids used in our study, pCI-SRC, pCI-SRC Y530F, and pCI-SRC K298M, were generated using QuickChange® site-directed mutagenesis (Stratagene, La Jolla, CA) and have been described previously [34]. The ING1b plasmid pCI-ING1b had been constructed as described previously [41]. For plasmid transfections, lipofectamine (Invitrogen, CA) was used following the manufacturer’s protocols.

### *In vitro* Kinase Assay

ING1 and Src proteins were purified as described [26], [56]. Purified baculovirus-expressed human Src kinase from our lab is also available commercially (Millipore, Billerica, MA, product 14–117). To perform the kinase assay, purified ING1 was incubated with purified Src in kinase buffer (50 mM HEPES pH 7.8, 5 mM MgCl_2_, 150 mM NaCl, 1 mM DTT, 1 mM ATP) plus inhibitors (1 mM sodium orthovanadate, 4mg/ml p-nitrophenyl phosphate) at 30°C for 30 minutes. The reactions were stopped with the addition of Laemmli sample buffer before being subjected to gel electrophoresis and western blotting.

### Immunoprecipitation and Western Blotting

Cell extracts in NP40 lysis buffer (150 mM NaCl, 50 mM Tris pH 7.5, 1% Nonidet p-40, 2 mM EDTA) supplemented with protease inhibitors (50 µg/ml leupeptin, 10 µg/ml aprotinin, 1 mM sodium orthovanadate, 4 mg/ml p-nitrophenyl phosphate) were immunoprecipitated with α-Src or α-ING1 antibodies for 2 hours at 4°C and then incubated for 1 hour at 4°C with a mixture of protein A and protein G beads. After immunoprecipitation, protein beads were washed four times with NP40 buffer and resuspended in sample buffer before gel electrophoresis and western blotting. BSA was used as a non-specific binding blocking agent, except for results shown in Fig. 1B in which powdered non-fat milk was used. Mab327 anti-Src antibody was a kind gift from Joan Brugge. Anti-α-tubulin mouse monoclonal antibody was purchased from Calbiochem (Calbiochem, NJ). Anti-phospho-tyrosine antibody 4G10 was provided by Steve Robbins. Anti-ING1 antibody was generated from hybridoma supernatant as described [57]. For western blotting, proteins were detected with the indicated primary antibodies followed by a species-specific secondary antibody conjugated with horseradish peroxidase. The protein bands were detected with ECL reagent (GE Healthcare, Backinghamshire, UK).

### Cycloheximide Block and Time Course Experiment

Cells were transfected as described above. Twenty-four hours post-transfection, cells were incubated with 100 µM cycloheximide (Sigma) for 0 and 8 hours before cell lysis and western blotting. The protein bands were scanned and quantified with a STORM 860 PhosphoImager (Molecular Dynamics, Sunnyvale, CA).

### Src Kinase and Specific Activity

Cells were lysed in RIPA buffer (50 mM Tris-Cl pH 7.2, 0.15 M NaCl, 1.0 M EDTA, 0.1% SDS, 1.0% Triton X-100, 1.0% sodium deoxycholate) supplemented with phosphatase and protease inhibitors (1 mM sodium orthovanadate, 3 mg/ml p-nitrophenolphosphate, 50 µg/ml leupeptin and 10 µg/ml aprotinin). c-Src was immunoprecipitated from the cell extracts with 327 anti-Src antibody (1 µg of antibody per 100 µg cell extract) for 1 hour at 4°C followed by incubation with 40 µl of protein A and protein G bead mix (1∶1 ratio) for 1 hour at 4°C. The beads were then washed 4 times with RIPA buffer and once with Src dilution buffer (50 mM Hepes pH 7.8, 150 mM NaCl, 1 mM DTT, 5 mM MgCl_2_, 200 µM sodium vanadate, 4 mg/ml p-nitrophenolphosphate). 50 µl of kinase assay buffer (Src dilution buffer containing 30 µM ATP, 1 µCi γ[^32^P]ATP (3000 Ci/mmol) and 100 µM Src optimal peptide was added to the immunoprecipitates and incubated for 15 minutes at 30°C. The reaction was stopped with 25 µl of 50% (v/v) acetic acid, after which 50 µl of the reaction mix was spotted on to a square of p81 phosphocellulose paper. The filter papers were then washed 5 times with 0.425% phosphoric acid, rinsed once with acetone and air dried before scintillation counting. To determine the specific activity of Src, a western blot of Src was performed on a duplicate set of Src immunoprecipitates from the same lysates, and the resulting bands were scanned and quantified with a STORM 860 PhosphoImager. The specific activity of Src (counts incorporated/band intensity) of each cell line represented is relative to the specific activity for SK-BR-3.

### Subcellular Fractionation

Cells were fractionated using the REAP method [38]. Briefly, HEK 293 cells transfected with the various pCI plasmids described above, were washed in ice cold phosphate buffered saline (PBS), detached from the dish with a cell scraper and collected in 1 ml of ice cold PBS. After centrifugation and removal of the supernatant, the cells were resuspended in 900 µl ice cold lysis buffer (0.1% NP40 in PBS) and triturated 5 times. 300 µl of the cells were set aside for whole cell lysate and the remaining 600 µl was centrifuged for 10 seconds. The supernatant (cytosolic fraction) was set aside and the pellet was resuspended in 600 µl of lysis buffer, triturated 5 times, and centrifuged for 10 seconds. This second supernatant of lysis buffer was discarded and the pellet was resuspended in 600 µl of lysis buffer (nuclear fraction). For each sample, equal volumes of whole cell lysate, cytosolic fractions and nuclear fractions were boiled in sample buffer before electrophoresis and western blotting.

### Apoptosis Assay

Exponentially growing cells were transfected with the various constructs as indicated for 24 hours and the degree of apoptosis was estimated by measuring sub-G1 DNA content with flow cytometry as described previously [47].

## Supporting Information

Figure S1 **Potential sites of ING1 phosphorylation as predicted by the NetPhos 2.0 (italics) KinasePhos (bold) and Motif Scan (underlined) programs. A)** Residues marked by asterisks have been previously reported to be phosphorylated. **B)** Tyrosine residues, predicted catalytic kinases and probability of the site being phosphorylated by the kinase using hidden Markov models (E-values are from KinasePhos, NP2 scores are from NetPhos 2.0 and Motif scores are from Motif Scan). Tyrosines 55 and 212 show the highest probability scores for being direct Src substrates although there is variability between program predictions.
(TIF)
