# Supplementary figures and images for: Src Regulates the Activity of the ING1 Tumor Suppressor
Source: PLoS One. 2013 Apr 9;8(4):e60943. doi: 10.1371/journal.pone.0060943 (PMC3621671; doi:10.1371/journal.pone.0060943)

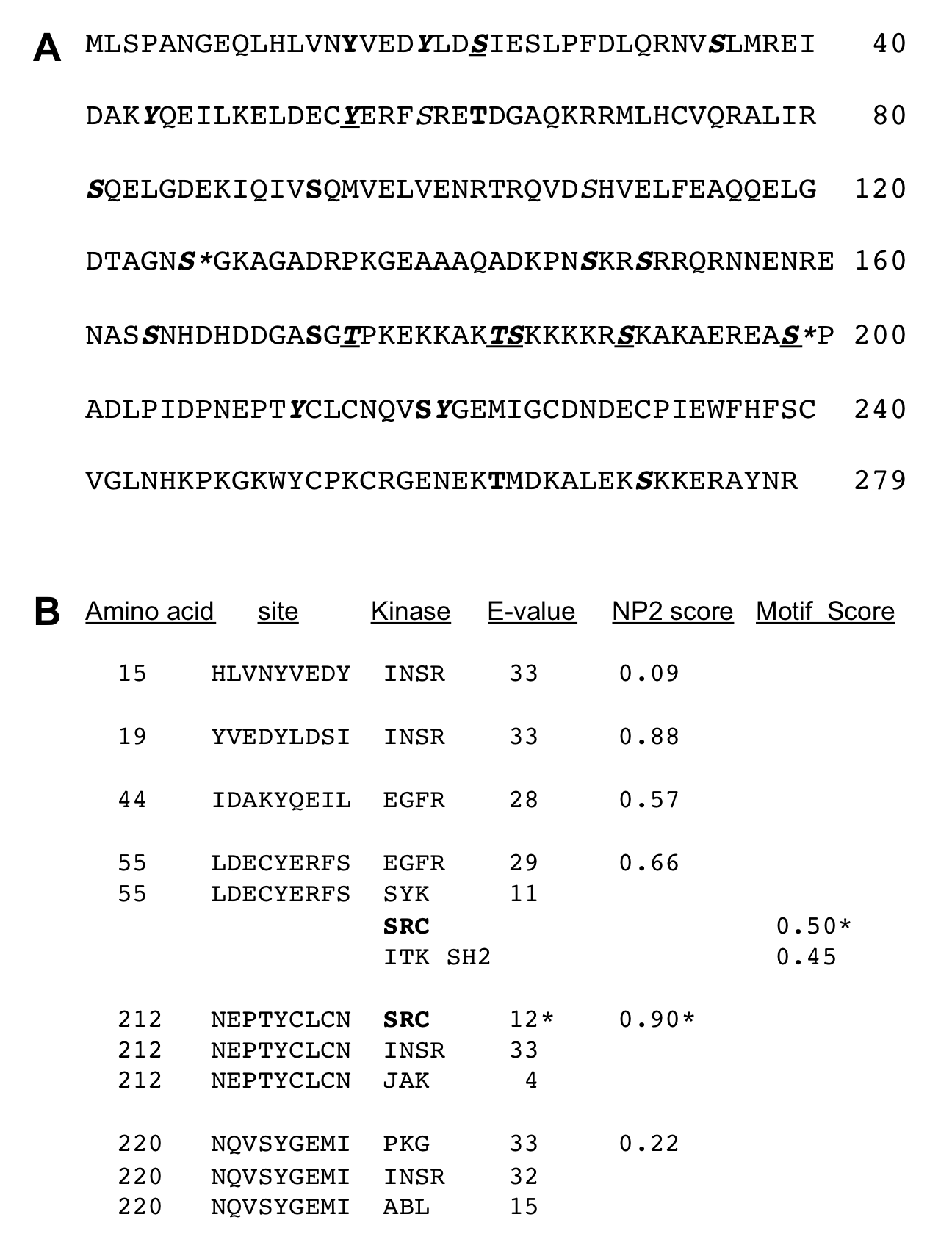

Supplement: Figure S1 — Potential sites of ING1 phosphorylation as predicted by the NetPhos 2.0 (italics) KinasePhos (bold) and Motif Scan (underlined) programs. A) Residues marked by asterisks have been previously reported to be phosphorylated. B) Tyrosine residues, predicted catalytic kinases and probability of the site being phosphorylated by the kinase using hidden Markov models (E-values are from KinasePhos, NP2 scores are from NetPhos 2.0 and Motif scores are from Motif Scan). Tyrosines 55 and 212 show the highest probability scores for being direct Src substrates although there is variability between program predictions. (TIF) [file pone.0060943.s001.tif]
